# Assessment of the effects of direct/video-observed therapy on pulmonary function test parameters and asthma control test: A single-center prospective randomized pilot study

**DOI:** 10.12669/pjms.42.7.13048

**Published:** 2026-07

**Authors:** Omer Selim Unat, Damla Serce Unat

**Affiliations:** 1 Omer Selim Unat Department of Pulmonology, Health Sciences University, Izmir Dr Suat Seren Chest Diseases and Surgery Training and Research Hospital, Türkiye; 2 Damla Serce Unat Department of Pulmonology, Bakircay University, Faculty of Medicine, Cigli Training and Research Hospital, Izmir; Türkiye

**Keywords:** Asthma, Inhaler Technique, Observed Therapy, Treatment Adherence, Video Consultation

## Abstract

**Background & Objective:**

Asthma control depends largely on adherence to inhaled controller therapy and correct inhaler technique, yet both remain suboptimal in routine practice. Telemedicine-based monitoring has emerged as a feasible strategy to support patient education and adherence. This pilot study aimed to evaluate the feasibility and potential effects of video-observed therapy (VOT) on pulmonary function test parameters and Asthma Control Test (ACT) scores in adults with asthma.

**Methodology:**

This single-center, randomized prospective pilot study was conducted between January 2024 and August 2024 at Giresun Dr. Ali Menekşe Chest Diseases Hospital on asthma patients who were not in an exacerbation phase. Participants were allocated to either a video-observed therapy (VOT) group or a control group using simple randomization. Patients in the VOT group received baseline inhaler training and were followed via brief (~3-minute) video calls on days 15 and 30. All patients were reassessed in person at day 45 with spirometry and ACT.

**Results:**

Eighty-eight patients were analyzed (VOT, n = 25; control, n = 63). At baseline, the VOT group had a lower ACT score than the control group (14.9 ± 1.9 vs 17.5 ± 3.7; *p* < 0.001). At day 45, the VOT group showed a significant within-group increase in FEV_1_ (+199 mL, +6.4%; *p* = 0.002), whereas the control group did not. FEF_25–75_ improved within both groups (*p* = 0.005 and *p* = 0.026, respectively), and ACT improved by approximately 3.5 points in each group (*p* < 0.001 for both). After ANCOVA adjusting for baseline ACT, the between-group effect on final ACT was borderline (*p* = 0.0501). In multivariable linear regression, VOT was not an independent predictor of change in FEV_1_ (β = 2.26, *p* = 0.62).

**Conclusion:**

This pilot study suggests that brief, structured video-observed therapy is feasible in routine outpatient asthma care and may support modest improvements in spirometric and patient-reported outcomes. However, adjusted analyses did not confirm an independent treatment effect, and these findings should be regarded as hypothesis-generating. Larger, multicenter randomized trials with balanced allocation, longer follow-up, and objective adherence and inhaler-technique measures are needed before video-observed therapy can be recommended for routine asthma management.

## INTRODUCTION

Asthma is a chronic airway inflammatory disease with a variable and heterogeneous course, typically presenting with dyspnea, wheezing, chest tightness, and cough. Its clinical phenotypes vary widely depending on the underlying inflammation type and etiology.^1^ Asthma maintenance follows a stepwise approach centered on inhaled corticosteroids (ICS). For patients uncontrolled on high-dose ICS plus long-acting beta-agonists, phenotype-driven biologics are used according to the inflammatory profile (eosinophilic or non-eosinophilic), such as anti-IgE and anti-IL-5. Long-acting muscarinic antagonists or oral corticosteroids may be added when needed.^2^

The principal goals of asthma management—preventing exacerbations, limiting airway remodeling, and minimizing treatment-related harm—depend critically on adherence to controller therapy and on correct inhaler use. Current GINA guidance recommends that adherence and inhaler technique be assessed at every clinical encounter.^3^ Despite this, real-world adherence to controller therapy is typically around 50%, and incorrect inhaler use is reported in roughly 39–65% of patients; both are independently associated with worse asthma control, lower lung function, and more frequent exacerbations.^4^

Observed therapy—originally developed for tuberculosis to ensure consistent medication intake—provides a conceptual framework for supporting adherence in other chronic respiratory diseases.^5^ Advances in mobile and video communication now make video-observed therapy (VOT) feasible in outpatient pulmonary care. Telemedicine has shown promising results for asthma follow-up, with effectiveness reported to be comparable to in-person visits in selected populations, and gained particular relevance during the COVID-19 era when standard pulmonary function testing and in-person education were disrupted.^6^,^7^ A recent pulmonology outpatient experience from Türkiye reported that video-based telemedicine was feasible in routine practice and that the majority of patients followed in that cohort had asthma, providing additional rationale for asthma-focused telemedicine studies in this setting.^8^

Evidence specifically evaluating brief, structured VOT combined with concurrent spirometric assessment in adult asthma remains limited, particularly from real-world outpatient settings. This pilot study aimed to evaluate the feasibility and potential effects of a brief video-observed therapy intervention on pulmonary function parameters and ACT scores in adults with asthma, in order to generate preliminary effect estimates that can inform the design of larger multicenter trials.

## METHODOLOGY

This single-center, prospective, randomized pilot study was conducted between January 2024 and August 2024 at Giresun Dr. Ali Menekşe Chest Diseases Hospital, a secondary-level governmental state hospital in Türkiye, where both authors were working during the study period, and enrolled adult asthma patients without acute exacerbation at presentation to the pulmonology clinic. Participants were randomly assigned to either an observed therapy or control group using simple randomization.

### Ethical approval:

Institutional Ethics Committee Approval No: 25.12.2023/02; dated December 27, 2023. Written informed consent was obtained from all participants.

### Inclusion criteria:

- Adults aged 18–75 years with asthma diagnosis confirmed by a pulmonologist;
- No acute exacerbation in the preceding 15 days, no airway obstruction on examination, and no need for systemic corticosteroids or antibiotics at baseline;
- Access to a mobile device capable of video calls (intervention group) ;
- Willingness to participate and ability to perform reliable spirometry.

### Exclusion criteria:

- Unconfirmed asthma diagnosis;
- Significant comorbidities (e.g., heart failure, chronic obstructive pulmonary disease, recent pneumonia, chronic kidney disease);
- Neurological or cognitive impairment limiting communication;
- Refusal to participate.

### Withdrawal Criteria:

- Patients who voluntarily requested to leave the study;
- Patients who did not respond to scheduled video follow-up calls;
- Patients who did not attend the scheduled control visit.

### Randomization and allocation:

Eligible patients were allocated to either the video-observed therapy (VOT) group or the control group using simple randomization. A fixed 1:1 allocation ratio was not pre-specified for this pilot study; the unequal final group sizes (VOT, n = 25; control, n = 63) reflect a combination of patient eligibility flow, patient preference for the less time-intensive arm, and follow-up loss. This imbalance is acknowledged as a limitation, and ANCOVA was used to adjust for the most clinically relevant baseline imbalance (ACT score).

### Intervention

All participants received identical baseline inhaler technique training from a respiratory nurse on the day of enrollment.

### VOT group:

In addition to baseline training, participants received structured video calls on day 15 and day 30. Each call lasted approximately three minutes; during each call, inhaler technique was reviewed visually, adherence was discussed, and patient-reported difficulties were addressed.

### Control group:

Participants received the same baseline training only, without scheduled mid-period reinforcement.

Both groups were reassessed in person at day 45 with repeat clinical evaluation, spirometry, and the ACT. The 45 days follow-up interval was selected as a pragmatic short-term window to capture early effects of the intervention on adherence-related outcomes, broadly consistent with the short follow-up schedules used in previous adherence-targeted asthma trials.^9^ No maintenance therapy changes were made during the study, and no agents capable of producing rapid pulmonary improvement (e.g., intravenous corticosteroids) were administered.

### Outcome measures:

Outcomes were within- and between-group changes from baseline to day 45 in spirometric parameters (FVC, FEV_1_, FEV_1_/FVC, FEF_25–75_; in mL and % predicted) and in ACT score. Inhaler technique and self-reported adherence were assessed at baseline only; they were not formally re-scored at follow-up. This is explicitly acknowledged as a limitation in the Discussion.

### Statistical analysis:

Analyses were performed using SPSS version 28.0 (IBM Corp., Armonk, NY). Distribution normality was assessed using the Shapiro–Wilk and Kolmogorov–Smirnov tests. Normally distributed variables are presented as mean ± standard deviation, and non-normally distributed variables as median (interquartile range). Between-group comparisons of baseline and follow-up variables were performed using independent-samples t-test or Mann–Whitney U test for continuous variables, and chi-square test for categorical variables. Within-group changes from baseline to follow-up were assessed using paired t-test or Wilcoxon signed-rank test. To adjust for the baseline imbalance in ACT, analysis of covariance (ANCOVA) was used with final ACT as the dependent variable, baseline ACT as the covariate, and group as the fixed factor. Multivariable linear regression was used to assess whether VOT independently predicted change in FEV_1_ after adjustment for age, sex, and smoking pack-years. Two-sided p values < 0.05 were considered statistically significant.

## RESULTS

### Patient flow and Baseline characteristics:

**Fig.1:**
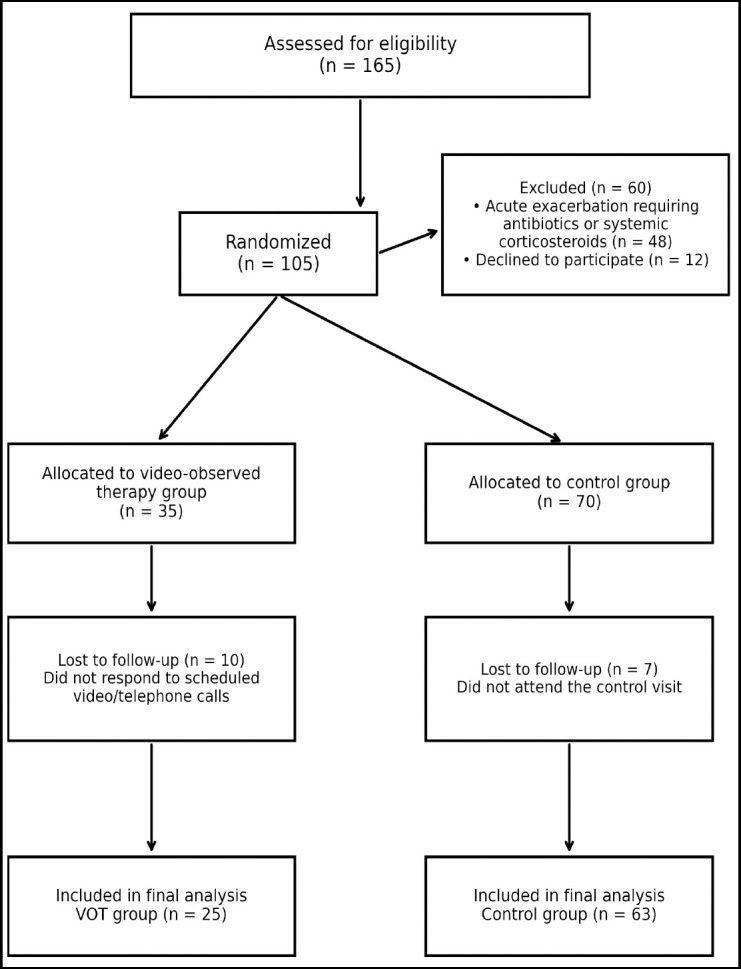
Flow Diagram.

A total of 88 patients were included in the final analysis (VOT, n = 25; control, n = 63). The CONSORT-style patient flow is shown in Fig.1. The mean age of the overall cohort was 55.3 ± 14.5 years, and 75% were women. A smoking history was reported by 31 patients (35.2%; 17 current, 14 former). Baseline demographics and most spirometric parameters were comparable between groups; however, the control group had higher mean pack-years (23.2 ± 9.6 vs 13.2 ± 5.1; *p* = 0.03) and a higher baseline ACT score (17.5 ± 3.7 vs 14.9 ± 1.9; *p* < 0.001). Full baseline and follow-up parameters are presented in Table-I.

**Table-I:** Clinical, demographic, and respiratory parameters between control and observed therapy groups.

Parameters	OT Group (n=25)	Control Group (n=63)	p value
Age*	55.2±12.3	55.4±15.4	0.96
Gender (female)*	22 (88)	44 (69.8)	0.10
No smoking history*	14 (56)	43 (68.3)	0.41
Ex-smoker*	5 (20)	12 (19)	
Active smoker*	8 (32)	6 (9.6)	
Smoking Pack-Years**	13.2±5.1	23.2±9.6	0.03
FEV_1_ (mL) initial**	2177.6±593.9	2171.3±778.5	0.97
FEV_1_ (%) initial**	82.9±18.2	78.6±19.7	0.33
FVC (mL) initial**	2738.4±731.8	2740.2±886.8	0.99
FVC (%) initial**	83.2±15.7	79.6±17.2	0.35
FEF_25-75_ (mL) initial**	2048±793.9	2143±1188.5	0.67
FEF_25-75_ (%) initial	88±35.1	78.9±39.4	0.30
ACT initial**	14.9±1.9	17.5±3.7	<0.001
FEV_1_ (mL) final**	2376.8±810.4	2182.2±768	0.21
FEV_1_ (%) final**	89.3±17.7	79.2±19.7	0.025
FVC (mL) final**	2900.8±677.8	2677±810.4	0.19
FVC (%) final**	87.1±15.2	77.8±14.6	0.012
FEF_25-75_ (mL) final**	2387.6±1097	2320.8±1287.6	0.78
FEF_25-75_ (%) final**	102.3±44.1	87.2±43.5	0.11
ACT final**	18.2±2.5	20.9±3.7	<0.001

* (n,%),

** (mean±SD).

### Within-Group changes:

Within-group changes from baseline to day 45 are presented in Table-II. In the VOT group, FEV_1_ increased significantly (+199 ± 289 mL; *p* = 0.002), as did FEV_1_ % predicted (+6.4 ± 10.4%; *p* = 0.002), whereas these parameters did not change significantly in the control group. FEF_25–75_ improved significantly in both groups (VOT: +350 mL, *p* = 0.005; control: + 178 mL, *p* = 0.026). ACT scores increased significantly in both groups, with a similar magnitude of change (VOT: +3.7 ± 2.5 points; control: +3.4 ± 4.1 points; *p* < 0.001 for both).

**Table-II:** Differences between initial and final parameters in the observed therapy and control groups.

Parameters	OT Group (n=25)	Control Group (n=63)
FEV_1_ (mL) difference between initial and final *	+199.2±288.8 (p=0.002)	+11.0±225.6 (p=0.70)
FEV_1_ (%) difference between initial and final *	+6.4±10.4 (p=0.002)	+0.7±10.3 (p=0.59)
FVC (mL) difference between initial and final *	+162.4±503.4 (p=0.120)	-63.2±393.4 (p=0.21)
FVC (%) difference between initial and final *	+3.9±14.2 (p=0.185)	-1.8±12.2 (p=0.24)
FEF_25-75_ (mL) difference between initial and final *	+349.6±562.9 (p=0.005)	+177.8±619.7 (p=0.026)
FEF_25-75_ (%) difference between initial and final *	+14.2±21 (p=0.002)	+8.3±22.8 (p=0.005)
ACT difference between initial and final *	+3.7±2.5 (p<0.001)	+3.4±4.1 (p<0.001)

* (mean±SD).

### Between-Group differences at Follow-up:

**Fig.2:**
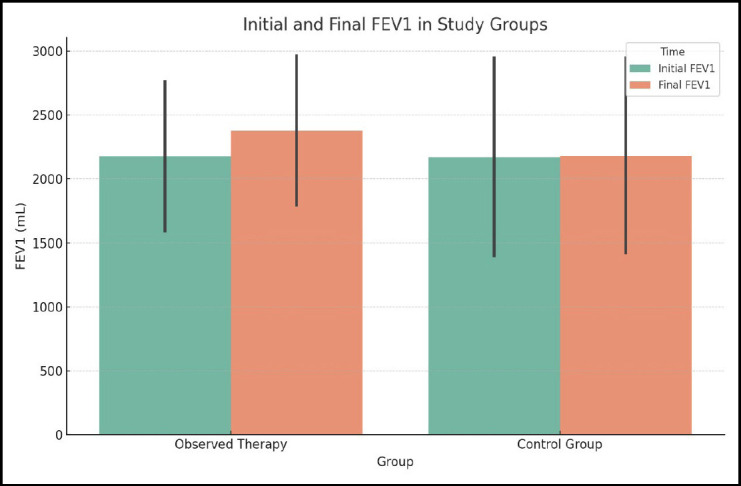
Comparison of initial and final FEV_1_ values between control group and observed therapy group.

At day 45, absolute FEV_1_ and FVC values did not differ between groups, but predicted values favored the VOT group: FEV_1_ % predicted (89.3 ± 17.7 vs 79.2 ± 19.7; *p* = 0.025) and FVC % predicted (*p* = 0.012). The final ACT score remained higher in the control group, mirroring the baseline difference (20.9 ± 3.7 vs 18.2 ± 2.5; *p* < 0.001). The trajectory of FEV_1_ from baseline to follow-up in both groups is illustrated in Fig.2.

### Adjusted analyses:

Because baseline ACT differed significantly between groups, an ANCOVA was conducted with final ACT as the dependent variable, baseline ACT as the covariate, and group as the fixed factor (Table-III). Baseline ACT was a strong predictor of final ACT (*p* = 0.0002), whereas the between-group effect was borderline (*p* = 0.0501). In multivariable linear regression with ΔFEV_1_ as the dependent variable, VOT was not an independent predictor (β = 2.26, *p* = 0.62) after adjustment for age, sex, and pack-years. Age showed a borderline association with ΔFEV_1_ (β = 0.30, *p* = 0.09) (Table-IV).

**Table-III:** Ancova Results for Final ACT Score.

Variable	Sum of Squares	df	F	p-value
Group	39.32	1	3.95	0.0501
Initial ACT	151.04	1	15.17	0.0002
Residual	846.50	85	-	-

**Table-IV:** Multivariable Linear Regression Analysis for ΔFEV_1_ (mL).

Variable	β Coefficient	Std. Error	t	p-value
Intercept	-12.98	8.85	-1.47	0.1546
Observed Therapy (Group)	2.26	4.48	0.50	0.6189
Age	0.30	0.17	1.76	0.0908
Male Sex	-6.35	5.07	-1.25	0.2215
Smoking (Pack-Years)	0.016	0.22	0.07	0.9415

## DISCUSSION

In this single-center pilot study, video-observed therapy was feasible to deliver in a routine outpatient pulmonology setting and was associated with within-group improvements in FEV_1_, FEV_1_ % predicted, FEF _25_–_75_, and ACT score over 45 days. The control group also showed gains in FEF_25–75_ and ACT but did not improve significantly in FEV_1_. However, in adjusted analyses, the between-group effect on final ACT was only borderline (*p* = 0.0501), and VOT was not an independent predictor of ΔFEV_1_ in multivariable regression. These findings should therefore be interpreted as preliminary and hypothesis-generating rather than confirming an independent treatment effect.

Suboptimal adherence to controller therapy and incorrect inhaler technique remains among the most consistent and modifiable barriers to asthma control. A systematic review by Engelkes and colleagues showed that good adherence was associated with significantly fewer severe exacerbations, identifying adherence as a clinically meaningful target.^10^ Despite decades of educational efforts, however, real-world adherence has not appreciably improved, and clinicians often struggle to convert recognition of this problem into sustained behavioral change at the individual patient level.^11^ A long-term systematic review of inhaler-use studies by Sanchis et al. concluded that approximately half of patients used their inhalers incorrectly and that this proportion had not improved over the preceding four decades.^12^ Improper inhaler technique has been independently linked to poor asthma control and increased emergency department visits,^13^ and in a Turkish tertiary-hospital sample Çil et al. similarly reported a high rate of inhaler misuse among asthma patients with a corresponding association with worse clinical and spirometric outcomes, suggesting that the same problem observed globally is also relevant in our regional setting.^14^ Interventions to improve inhaler technique have been examined in a Cochrane review, which found that face-to-face physical training or repeated demonstration can produce short-term improvements in technique, although effects on clinical outcomes such as exacerbations and asthma control were inconsistent.^15^ Active behavioral interventions such as electronic dose reminders have likewise been shown to increase controller-medication adherence in primary-care asthma populations, although effects on lung function or exacerbations have been more variable.^16^ Our observation of larger within-group FEV_1_ gains in the VOT arm is broadly compatible with this body of evidence; however, because inhaler technique and adherence were not objectively re-measured at day 45, the spirometric and ACT improvements cannot be directly attributed to behavioral change, and other contributors such as regression to the mean and the Hawthorne effect cannot be excluded.

Telemedicine has emerged as a practical strategy to extend asthma education and follow-up beyond the clinic. Portnoy et al. demonstrated that synchronous telemedicine visits were as effective as in-person consultations in selected asthma populations,^6^ and the systematic review and meta-analysis by Chongmelaxme et al. concluded that telemedicine-based interventions can modestly improve asthma control and quality of life in adults compared with usual care.^17^ The Cochrane review by Kew and Cates similarly found that remote check-ups for asthma did not significantly differ from face-to-face care in terms of exacerbations, asthma control, or quality of life, supporting non-inferiority of remote follow-up in selected groups while also highlighting that benefits on hard clinical outcomes remain modest and uncertain.^18^ A broader meta-review of supported self-management for asthma further emphasized that personalized action plans and structured professional support — components that map closely onto our brief video-call format — are associated with reductions in exacerbations and improvements in quality of life.^19^ During the COVID-19 era, virtual care additionally helped maintain continuity in pulmonary follow-up when standard laboratory-based pulmonary function testing was disrupted.^7^ In a directly relevant local experience, Çetin et al. described videoconference-based telemedicine in a respiratory diseases outpatient clinic in Türkiye and reported that asthma was among the most frequent diagnoses managed in this way, supporting the feasibility of videoconferencing in routine outpatient respiratory care in our regional setting.^8^ Our intervention used a deliberately minimal format — two brief, approximately three-minute video calls at days 15 and 30 — that fits readily into a real-world outpatient workflow without requiring dedicated telemedicine infrastructure.

What this study adds is preliminary, prospective evidence on the feasibility of a brief, structured VOT intervention combined with objective spirometric reassessment in adult asthma patients in Türkiye, complementing existing predominantly retrospective and questionnaire-based telemedicine work. Although the adjusted between-group differences were not robust, the within-group improvements in the VOT arm provide effect-size estimates that can inform sample-size calculations for definitive trials. The simultaneous improvement in FEF_25–75_ in both groups, recognized as a small-airway marker that may capture treatment response beyond FEV_1_, is in line with the wider recognition that small-airway involvement is clinically relevant in asthma monitoring.^20^ Clinically, brief scheduled video check-ins may represent a low-cost adjunct to routine asthma care that warrants further structured evaluation, particularly in settings where dedicated telemedicine platforms are not available, but they cannot yet be recommended as a stand-alone strategy on the basis of these data.

### Strengths

Strengths of this study include its prospective design; its real-world outpatient setting; the combined use of subjective (ACT) and objective (spirometric) outcomes; the application of adjusted statistical models (ANCOVA and multivariable regression) rather than relying solely on within-group changes; and the simplicity and low resource intensity of the intervention, which favors potential implementation in settings with limited telemedicine infrastructure.

### Limitations

Several limitations should be acknowledged. First, this was a single-center pilot study with a relatively small overall sample and unequal group sizes, both of which reduce statistical power and limit generalizability. Second, a fixed allocation ratio was not pre-specified, contributing to the unequal group sizes and to a baseline ACT imbalance; although ANCOVA was used to adjust for baseline ACT, residual confounding cannot be excluded, and an additional imbalance-adjusted analysis (e.g., propensity-score adjustment) was not feasible given the available dataset and the pilot scope of the study. Third, blinding of participants and outcome assessors was not feasible. Fourth, although baseline inhaler-technique training was identical in both groups, inhaler technique and adherence were not objectively re-evaluated at day 45 using a standardized checklist or electronic monitoring; consequently, the contribution of behavioral change to the observed spirometric and ACT improvements cannot be directly quantified, and this represents a major limitation. Fifth, follow-up was limited to 45 days, so the durability of any benefit is unknown. Finally, multivariable regression did not confirm an independent association between VOT and ΔFEV_1_, indicating that any observed advantage may be partly explained by other patient factors.

## CONCLUSION

This pilot study suggests that a brief, structured video-observed therapy intervention is feasible in real-world outpatient asthma care and is associated with modest within-group improvements in spirometric parameters and asthma control. However, adjusted analyses did not confirm an independent treatment effect, and these findings should be regarded as hypothesis-generating. Larger, well-balanced, multicenter randomized controlled trials with objective adherence measures and longer follow-up are needed before video-observed therapy can be recommended for routine asthma management.

### Future directions:

Future studies should be designed as larger, multicenter randomized controlled trials with pre-specified balanced allocation; blinded outcome assessment where feasible; longer follow-up to assess durability and exacerbation outcomes; objective adherence measures such as electronic dose-monitoring devices or prescription-refill data; and standardized inhaler-technique scoring using validated checklists. Patient-reported satisfaction, acceptability, and feasibility outcomes should be assessed alongside clinical endpoints to inform real-world implementation in pulmonology outpatient practice. Embedding such video-based interventions within established self-management frameworks, rather than testing them as stand-alone tools, may also better reflect how telemedicine is likely to be deployed in routine asthma care.

### Use of AI Tools:

Artificial-intelligence-assisted language editing was used during the preparation of this manuscript to improve grammar and clarity of expression. All scientific content, data analysis, interpretation of results, and final responsibility for the manuscript remain solely with the authors.

### Authors’ Contribution:

**OSU:** Conceptualization of the study, patient recruitment, video consultations, data collection, and manuscript drafting.

**DSU:** Patient recruitment, data collection, data analysis, statistical interpretation, and critical revision of the manuscript.

Both authors approved the final version of the manuscript and agreed to be accountable for all aspects of the work in ensuring its accuracy and integrity.
